# Advancing on-chip Kerr optical parametric oscillation towards coherent applications covering the green gap

**DOI:** 10.1038/s41377-024-01534-x

**Published:** 2024-08-21

**Authors:** Yi Sun, Jordan Stone, Xiyuan Lu, Feng Zhou, Junyeob Song, Zhimin Shi, Kartik Srinivasan

**Affiliations:** 1 Microsystems and Nanotechnology Division, Physical Measurement Laboratory, National Institute of Standards and Technology, Gaithersburg, MD USA; 2 Joint Quantum Institute, NIST/University of Maryland, College Park, MD USA; 3 Reality Labs Research, Meta, Redmond, WA USA

**Keywords:** Microresonators, Nonlinear optics, Silicon photonics, Integrated optics, Nanophotonics and plasmonics

## Abstract

Optical parametric oscillation (OPO) in Kerr microresonators can efficiently transfer near-infrared laser light into the visible spectrum. To date, however, chromatic dispersion has mostly limited output wavelengths to >560 nm, and robust access to the whole green light spectrum has not been demonstrated. In fact, wavelengths between 532 nm and 633 nm, commonly referred to as the “green gap”, are especially challenging to produce with conventional laser gain. Hence, there is motivation to extend the Kerr OPO wavelength range and develop reliable device designs. Here, we experimentally show how to robustly access the entire green gap with Kerr OPO in silicon nitride microrings pumped near 780 nm. Our microring geometries are optimized for green-gap emission; in particular, we introduce a dispersion engineering technique, based on partially undercutting the microring, which not only expands wavelength access but also proves robust to variations in resonator dimensions. Using just four devices, we generate >150 wavelengths evenly distributed throughout the green gap, as predicted by our dispersion simulations. Moreover, we establish the usefulness of Kerr OPO to coherent applications by demonstrating continuous frequency tuning (>50 GHz) and narrow optical linewidths (<1 MHz). Our work represents an important step in the quest to bring nonlinear nanophotonics and its advantages to the visible spectrum.

## Abstract (web summary)

Kerr optical parametric oscillation in silicon nitride microrings pumped near 780 nm generates > 150 wavelengths across the green gap (532-633 nm) with <1 MHz linewidths using only four devices.

## Graphical abstract

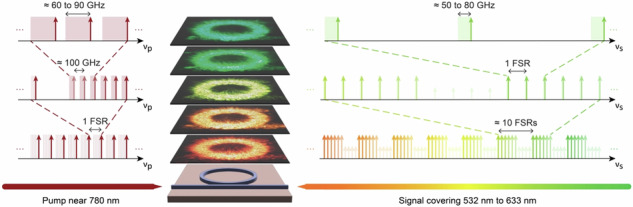

## Introduction

**Fig. 1:**
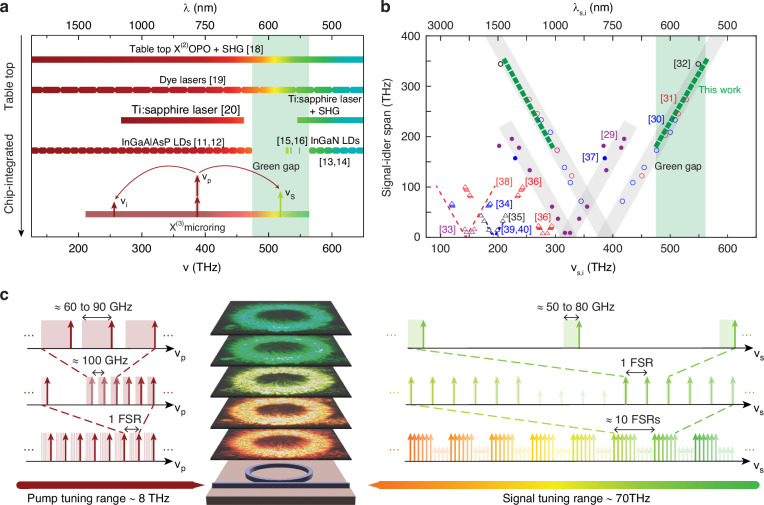
Aiming for the green gap with Kerr microresonator optical parametric oscillation (OPO). **a** Comparison of currently available laser technologies. Table-top OPO and second harmonic generation (SHG) provide broad spectral coverage using bulk free-space optics^18^. Commercial dye lasers, which are also table-top technologies, rely on potentially toxic chemicals and face a limitation in that each dye only offers a tuning range of 50 nm to 100 nm, necessitating multiple dyes to achieve a broader range of tuning capabilities^19^. Ti:sapphire lasers are highly tunable and have recently been demonstrated on-chip^20^, but they exhibit (even when combined with SHG) a large spectral gap for orange, yellow, and green colors. Commercially-available laser diodes can emit a wide range of infrared and visible wavelengths, but the active region (which is composed in tunable ratios of two materials) emission spectrum exhibits a gap between 532 nm and 633 nm^11–14^ with few exceptions^15,16^. This gap has become known as the “green gap”. On the other hand, OPO in Kerr microresonators can generate orange, yellow, and green colors without spectral gaps. **b** Comparison of chip-integrated OPO results, charting demonstrated signal and idler frequencies (*ν*_s_ and *ν*_i_) and their separation for various χ^(2)^ and χ^(3)^ (Kerr) systems. While χ^(2)^ OPOs are mostly focusing on infrared wavelengths above 1400 nm shown in red and blue lines^38–40^, Kerr OPOs could span from infrared to the visible wavelengths, as indicated by the triangles and circles^29–37^. Data from this work (compiled from four separate devices) are indicated by the dashed green line, where the dashed line is used to acknowledge the existence of spectral gaps. **c** Illustration of three pump-actuated OPO tuning mechanisms. When the pump laser (frequency *ν*_p_) is switched between adjacent longitudinal modes, i.e., *ν*_p_ shifts by one free spectral range (FSR), ≈900 GHz, *ν*_s_ changes by ≈9 THz (bottom row). When *ν*_p_ is tuned within one cavity mode, thermo-optic effects induce changes to dispersion that cause *ν*_s_ to mode hop in FSR increments (middle row). In between mode hops, *ν*_s_ tunes continuously with *ν*_p_ with an approximately 6:5 ratio (top row), resulting in up to 80 GHz of tuning. The stacked color images are of scattered light for different signal wavelengths generated by the µOPOs in this work

The development of compact visible lasers will benefit numerous sectors of science and industry, including laser lighting and displays^1,2^, spectroscopy for timekeeping and sensing^3–5^, medical practices^6^, and quantum technology^7,8^. While progress has been made in the blue and red wavelength regions, a lack of efficient and compact green laser sources, also known as the “green gap” problem (Fig. 1a), still plagues the laser market^9,10^. III-V semiconductor lasers provide a compelling combination of efficiency and small size^11–14^, but they require Watts of input power and often (especially at “green gap” wavelengths) lack the spectral purity needed for high-coherence applications^15,16^. Injection locking Fabry-Pérot diode lasers to high-finesse microresonators can improve coherence, but the output wavelengths are constrained by the availability of pump lasers and, so far, are continuously tunable over only a few GHz^17^. In Fig. 1a, we compare various commercial solutions to the green gap problem^18–20^, charting them by their size and wavelength range.

Another way to produce green laser light is through nonlinear optical processes. This is the strategy adopted most by industry, and it offers an intriguing path to scalability via photonic integration since small optical volumes promote efficient nonlinear interactions (commercial instruments using bulk optical components are typically ≈1 m^3^ in size). For example, nonlinear microresonators can generate the frequency harmonics of near-infrared pump lasers to produce visible light, albeit with limited wavelength tuning capability^21–27^. Alternatively, widely-separated Kerr optical parametric oscillation (OPO) is a flexible approach to generate visible light by four-wave mixing (FWM) from, e.g., a near-infrared pump, and in recent years, OPO based on FWM in optical microresonators (we dub such devices “µOPOs”) has been investigated^28–37^. In these systems, energy from a monochromatic pump laser with frequency *ν*_p_ is transferred to a blue-shifted signal wave (*ν*_s_) and red-shifted idler wave (*ν*_i_), as shown in Fig. 1a. Visible µOPOs can operate with milliwatt-level threshold powers and have shown pump-to-sideband conversion efficiencies up to 15%^29,30^. In Fig. 1b, we compare the operating wavelengths and spectral separations, *ν*_s_-*ν*_i_, reported in several µOPO studies, including, for completeness, several demonstrations of OPO in χ^(2)^ nanophotonics^38–40^. Importantly, signal frequencies in the green spectrum have been reported^30–32^, but the highest frequency reported so far is ≈548.9 THz^32^, which is ≈14.6 THz shy from the edge of the green gap. In addition, the µOPO output power and wavelength are sensitive to external parameters like temperature, pump power, and pump-resonator detuning^41^, as well as to microring geometry. These sensitivities tend to grow in proportion to the µOPO separation (*ν*_s_−*ν*_i_) [ref. ^31^] and therefore present a major challenge for µOPOs aiming at more comprehensive coverage of the green gap.

Here, we use µOPOs to access the entire green gap, achieving the highest frequency of ≈563.51 THz, increasing wavelength access by ≈14.2 nm beyond the previous record in ref. ^32^ and improving robustness with respect to parameter variations. Using just four devices, we can selectively generate >150 µOPOs, each with a unique green-gap signal frequency that is separated from its nearest neighbor by roughly the microresonator free spectral range (FSR). This breakthrough is enabled by a novel dispersion design in which the substrate is partially etched away, so that a greater portion of the microresonator is air-clad. We perform simulations and measurements to explore the effects of such an undercut on the µOPO. In particular, increasing the undercut makes *ν*_s,i_ less sensitive to *ν*_p_ and device dimensions; hence, one device supports many green-gap µOPOs, and spectral gaps are filled in using a second device with different dimensions. Our ability to generate a multitude of µOPOs within a single device stems from unique tuning mechanisms (two mode hop-based processes for coarse tuning and one process for continuous fine tuning) that we depict in Fig. 1c. Finally, to prove our µOPOs are well-suited to coherent applications in the green gap, we present measurements of heterodyne beatnotes between the µOPO signal and a separate narrow-linewidth laser, and we characterize the µOPO continuous frequency tunability. We measure fitted linewidths below 1 MHz and continuous tuning ranges up to 80 GHz. With further integration, including recent advancements in chip-integrated 780 nm lasers^17,42,43^, µOPOs are a realistic solution to the green gap problem, especially when low noise is required.

## Results

### Device design

**Fig. 2:**
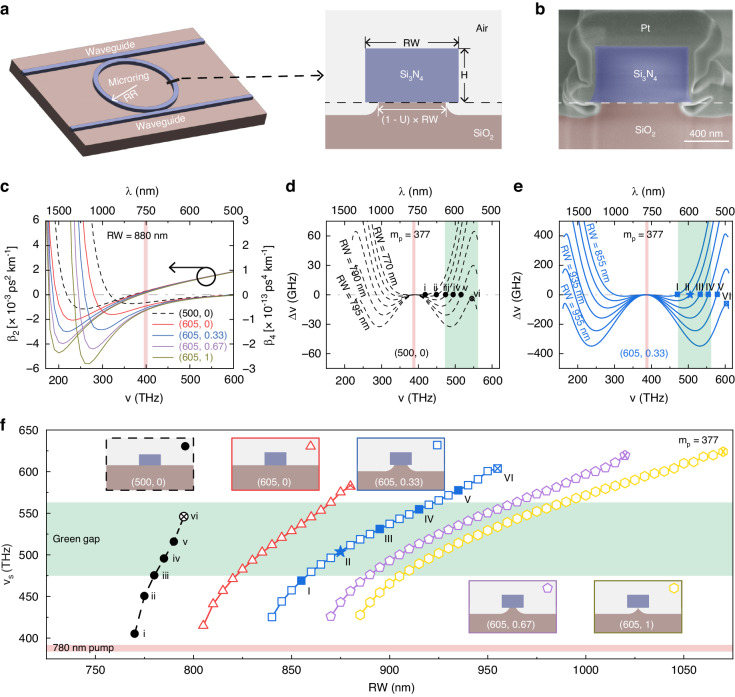
Microresonator dispersion design. **a** Left: Illustration of a microring OPO device, indicating the outer ring radius (RR) and two coupling waveguides that are used for separately extracting the signal and idler waves. Right: Illustration of the microring cross section. The device has a silicon nitride (Si_3_N_4_, hereafter SiN) core, a silicon dioxide (SiO_2_) substrate, and top air cladding. Its dimensions are defined as ring width (RW) and height (H). We also use potassium hydroxide (KOH) to etch the SiO_2_ substrate underneath the SiN core for dispersion engineering. The etch is quantified by the undercut parameter, U. **b** False-color cross-sectional scanning electron microscope (SEM) image from one device milled by focused ion beam. The noticeable “foot” region has a negligible impact on dispersion, as explored through simulations presented in Supplementary Information Fig. S2. **c** Simulations of the second- and fourth-order expansion coefficients (β_2_ and β_4_) of the TE0 mode propagation constant for different microring geometries (parameterized as (H, U) and RW = 880 nm). **d** Simulated frequency mismatch spectra for six devices with (500, 0) and RW ranging from 770 nm to 795 nm with increments of 5 nm. Points i-v mark zero crossings, which predict the signal frequency (*ν*_s_). **e** Simulated frequency mismatch spectra for six devices with (605, 0.33) and RW ranging from 855 nm to 955 nm with increments of 20 nm. **f** Simulated values of *ν*_s_ versus RW for different (H, U) designs. Points marked with numerals correspond to those displayed in **d** and **e**. Data points with crosses mark the upper limit for *ν*_s_. For **d**–**f**, the pump mode azimuthal number, m_p_, is fixed at 377. In general, thicker SiN increases higher-order dispersion to broaden the OPO spectral coverage. We find H = 605 nm is sufficient to cover the green gap. Furthermore, increasing U not only increases spectral coverage but also decreases the sensitivity of *ν*_s_ to RW, resulting in more robust green light generation and deeper control (e.g., via the tuning mechanisms described in Fig. 1) over the OPO spectrum. Point II, designated with a star symbol, corresponds to our experimental design

In Fig. 2, we present images of a nominal microring device as well as simulations of the chromatic dispersion. We fabricate microrings out of stoichiometric silicon nitride (Si_3_N_4_, hereafter written as SiN) with outer ring radius RR = 25 µm and nominal height H = 605 nm; the microrings sit on a SiO_2_ lower cladding and are air-clad on the sides and top (see “Materials and methods” section for details). We couple light in/out of the microrings via two bus waveguides, as shown in Fig. 2a. One waveguide is narrower and runs closer to the microring; it in/out-couples pump and signal light. The other waveguide is wider and farther from the microring and is used to out-couple the long-wavelength idler, which cannot propagate in the narrower waveguide (the waveguide is cut-off at the idler wavelength). Using heated potassium hydroxide, we undercut the microrings by an amount U that can be between 0 (no undercut) and 1 (completely undercut). In Fig. 2b, we show a scanning electron microscope image of the microring cross-section in which the undercut (U ≈ 0.25) is clearly visible.

We choose H and U to optimize dispersion, which we parameterize using the frequency mismatch, ∆*ν* = *ν*_µ_ + *ν*_−µ_ − 2*ν*_0_, where *ν*_µ_ is the frequency of a mode whose longitudinal mode number (with respect to the pump mode) is µ. In general, mode pairs with small positive ∆ν can oscillate^29–31^; hence, to realize µOPOs with wide frequency separations, we desire strong normal group velocity dispersion (GVD) in the pump band (negative curvature of ∆ν around *ν*_0_) and higher-order GVD to balance ∆ν away from *ν*_0_. To understand the relationships between H, U, and GVD, in Fig. 2c we present the simulated GVD coefficients, β_2_ and β_4_, for the fundamental transverse electric polarized (TE0) modes of five ring resonators with different (H, U) values. In the pump band, β_2_ is slightly positive (indicating normal dispersion) and nearly independent of H and U, while β_4_ becomes significantly more negative for increasing H and U (indicating greater higher-order dispersion that can balance the normal dispersion for mode pairs far from the pump). Moreover, we can make the general observation that the idler band (where β_2_ and β_4_ are more sensitive to ν, H, and U) primarily determines the geometric dispersion. Alternatively, we can study the ∆ν spectrum and its dependence on H and U. In Fig. 2d, e, we present six ∆*ν* spectra each for devices with (500, 0) and (605, 0.33), respectively, with systematic variations to RW. In the ∆*ν* space, zero crossings (marked in Fig. 2d, e by solid circles and squares, respectively) determine *ν*_s_, so we can predict specifically the dependence of *ν*_s_ on RW, H, and U. In Fig. 2f, we plot *ν*_s_ versus RW for several (H, U) pairs. We find that in the (500, 0) configuration that has been extensively used in prior studies^29–31^, *ν*_s_ is limited to less than 530 THz, and a relatively narrow range of RW values allow for green gap emission. However, increasing H and U has two notable effects: The maximum realizable *ν*_s_ is increased, and the µOPO is more robust to geometry perturbations. Specifically, the slope d*ν*_s_/dRW, which quantifies the µOPO sensitivity to device dimensions, decreases for increasing U. As a result, designs are more tolerant to fabrication uncertainties, thus improving device yields when targeting specific wavelengths. In Supplementary Information Fig. S1, we explore the sensitivity of *ν*_s_ to *ν*_p_ changes for different geometries and find that it decreases with increasing H or U, enabling more µOPOs from individual devices. We also note that, while H and U similarly impact the dispersion, thicker waveguides are difficult to etch and are vulnerable to cracking. In the next section, we experimentally verify these concepts and leverage them towards comprehensive access to the green gap.

### Green gap access

**Fig. 3:**
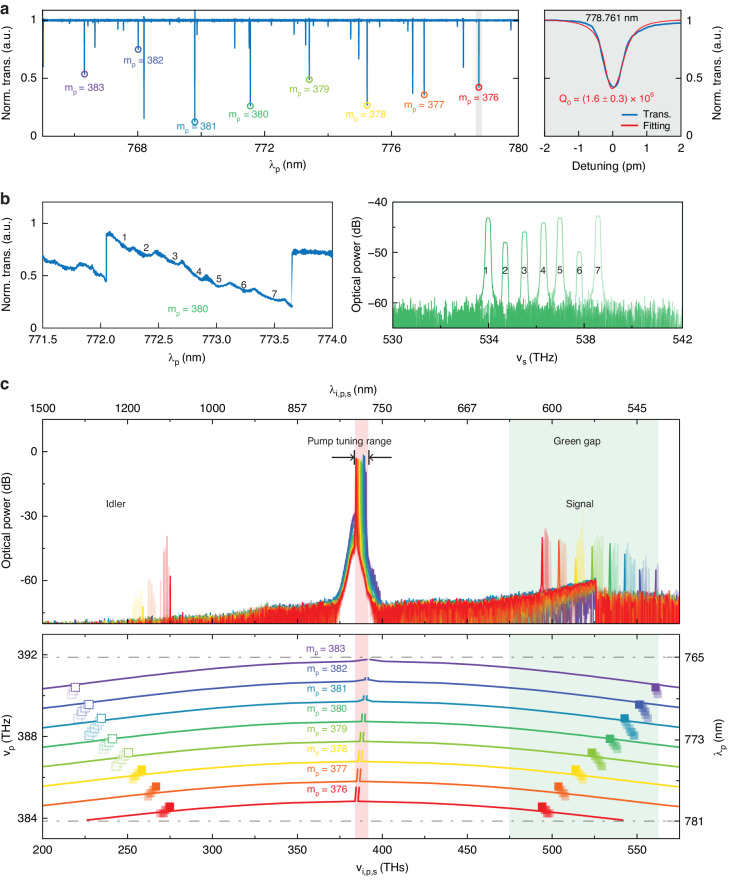
Green gap OPO and its coarse tuning. **a** Normalized pump-band transmission spectrum for a nominal µOPO device with H = 605 nm, U = 0.33, and RW = 875 nm. Eight TE0 modes (m_p_ = 376, …, 383) are marked with colored circles, and the fitted Q is shown in the right panel. The quoted uncertainty in Q is the one standard deviation value from the fit. **b** Left: Normalized high-power transmission for mode *m*_p_ = 380 of the device from **a**. We observe the characteristic “thermal triangle” when scanning *λ*_p_ from blue to red. Different detunings (marked by numerals 1 through 7) generate seven different µOPOs with *ν*_s_ differences of approximately one FSR. The corresponding signal spectra are shown in the right panel; here, 0 dB is referenced to 1 mW, i.e., dBm. **c** Top panel: Compilation of optical spectra generated by the nominal device. The different colors correspond to the *m*_p_ values indicated in **a**, and bold data correspond to the first OPO spectrum observed when the pump laser is blue-to-red scanned through a given pump mode. The faded data correspond to subsequently observed OPO spectra (e.g., spectra 2–7 in **b**). Idlers with frequencies below 250 THz are not observed due to the pump/signal access waveguide cutoff (Supplementary Information Fig. S3). Bottom panel: Distribution of the signal and idler frequencies versus simulations. Solid squares are experimental data extracted from the spectra above. Dashed empty squares are estimated based on energy conservation. Solid lines are taken from dispersion simulations

Figure 3 depicts the multitude of green gap µOPOs we make in experiments and illustrates the coarse tuning mechanisms that enable them. In our experiments, we pump TE0 modes in a SiN microring with an amplified external cavity diode laser (ECDL) that is continuously tunable from 765 nm to 781 nm. The measurement setup is illustrated in Supplementary Information Fig. S3. Figure 3a (left panel) depicts the normalized, low-power transmission spectrum of a device with RR = 25 µm, H = 605 nm, RW = 875 nm, and U = 0.33. The colored circles mark the eight TE0 modes we can access with the ECDL, and the right panel depicts the normalized transmission zoomed in to 778.761 nm (wavelength measured with a wavemeter with ≈0.1 pm uncertainty). When we increase the pump power, we can generate OPO, and we observe a characteristic “thermal triangle” shape in the transmission spectrum of each resonator mode when *ν*_p_ is scanned from blue to red detunings, as shown in the left panel of Fig. 3b. Moreover, for each specific m_p_ value (380 in Fig. 3b), adjusting the pump-resonator detuning allows us to tune *ν*_s_ in FSR-level increments through a mode-switching mechanism^41^, as shown by the seven different signal spectra presented in the right panel of Fig. 3b that correspond to the seven detunings marked in the left panel. The mode switching occurs due to changes in the effective dispersion that arise from Kerr- and thermal-nonlinear mode shifts. Interestingly, we believe that dispersion of the thermo-optic coefficient, d*n*/d*T*, where *n* is the refractive index of SiN and *T* is the modal temperature, must be considered to properly model the mode switching, but such measurements are not found in the existing literature.

To more widely tune *ν*_s_, we can pump modes with different m_p_. In the top panel of Fig. 3c, we present optical spectra compiled from a single microring, where each colorband corresponds to a different m_p_ value - stepping m_p_ by one shifts *ν*_s_ into a new colorband. Hence, the relatively small *ν*_p_ tuning range (≈8 THz) enables coarse *ν*_s_ tuning between ≈490 THz and ≈560 THz. This coarse tuning mechanism was reported in ref. ^31^ and arises from the *ν*_p_-dependent ∆*ν* spectrum. In the bottom panel, we chart the *ν*_s,i_ values extracted from the optical spectra above, and we compare our measurements to simulations. We find that simulations accurately predict the *ν*_s,i_ shifts that result from incrementing *m*_p_. The *ν*_p_ variation (for a given m_p_) that is evident in Fig. 3c is due to an underlying RW variation (i.e., moving along a line of constant *m*_p_ corresponds to varying RW). In Fig. 3c, bold data are associated with the first µOPO observed when *ν*_p_ is scanned into resonance from blue to red detunings, and faded data are associated with subsequent µOPOs observed during the scan. We typically observe between six and eight µOPOs for each m_p_ value, as shown in Fig. 3b.

### Tunability and coherence

**Fig. 4:**
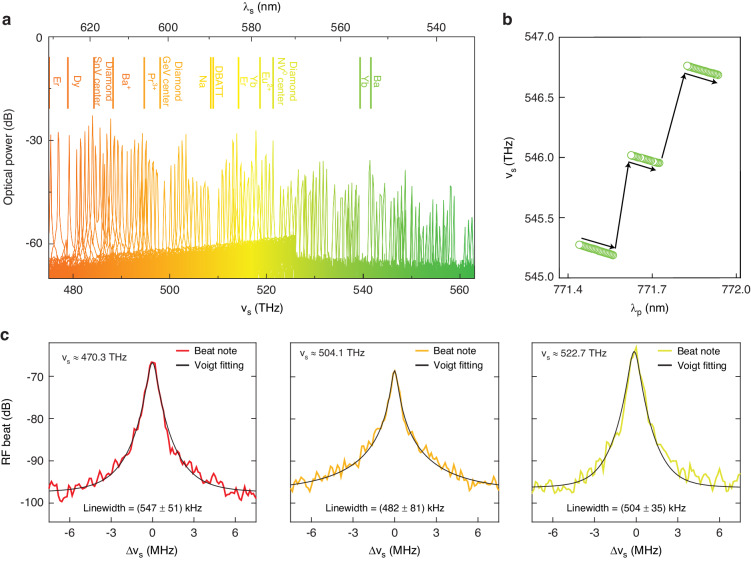
Continuous fine tuning of the signal frequency (ν_s_) and linewidth measurements. **a** Optical spectra compiled from four µOPO devices with small differences in RW. Frequencies greater than 490 THz are generated using only two devices. There are a few gaps and overlaps due to the fact that output spectra from these four devices are not perfectly matched. The geometry differences between the devices were not optimized to make their OPO spectra complementary, leading to these gaps and overlaps. Further optimizing the geometric differences between these devices can be another degree of freedom to maximizing the spectral coverage. The top vertical lines indicate transition wavelengths of various quantum systems within the green gap. **b** *ν*_s_ versus pump wavelength in a nominal µOPO device. Here, the pump wavelength is tuned within a single cavity resonance. Periodic mode hops occur that shift *ν*_s_ by approximately one FSR. In between mode hops, *ν*_s_ varies smoothly and continuously with *ν*_p_, with typical continuous tuning ranges between 50 GHz to 80 GHz. **c** Radiofrequency heterodyne beat notes between the OPO signal and a Ti:sapphire sum-frequency-generation laser system, along with corresponding fits and fit uncertainties. 0 dB is referenced to 1 mW

The spectra in Fig. 3c come from one device and exhibit spectral gaps in between colorbands. To address these gaps, we fabricate three more devices with small systematic RW differences. Detailed data for these devices are shown in Supplementary Information Fig. S4. In Fig. 4a, we present optical spectra compiled from this set of four devices that address the entire green gap with nearly FSR-level resolution. In particular, we use just two devices to generate more than 100 µOPOs, including all signal frequencies above 490 THz, and further design optimization could allow similar performance using only one device. For instance, using a larger RR will increase the density of longitudinal modes (and, in turn, the density of spectral coverage), employing chip-integrated heaters will allow thermo-optic dispersion control to actuate FSR-level *ν*_s_ tuning^44^, and the (H, U) design could be further optimized. Still, understanding the intertwined roles of these various “knobs” is a work in progress. We also note that the highest frequency achieved in our study, as detailed in Supplementary Information Fig. S3, is ≈563.51 THz, which is nearly 15 THz beyond the previous record^32^. Moreover, since many applications (e.g., spectroscopy of quantum systems - see marked level transitions in Fig. 4a) require lasers that are continuously tunable and phase coherent, we proceed to characterize the *ν*_s_ tunability and measure the µOPO linewidth. As described above, a useful coupling exists between *ν*_p_ and *ν*_s_, where small adjustments to the former (e.g., shifting the pump-resonator detuning) can induce FSR-level shifts in the latter. It is also crucial to understand the tuning dynamics in between such mode hops. In Fig. 4b, we present measurements of *ν*_s_, recorded using an optical spectrum analyzer (OSA), as *ν*_p_ is tuned in our nominal device. We observe that, in between mode hops, the tuning coefficient d*ν*_s_/d*ν*_p_ ≈ 1 (it is slightly greater than one due to thermo-optic shifts); and typically, we achieve continuous tuning ranges between 50 GHz and 80 GHz. Notably, this tuning range depends on the dispersion and can be extended using, e.g., integrated temperature control^44^, and we show some preliminary experimental results with temperature variation in Supplementary Information Fig. S5. We note that ‘bumpiness’ in Fig. 4b (i.e., any deviation from linear tuning) is primarily due to OSA error.

Next, we record radiofrequency spectra from heterodyne beats between the µOPO signal and a low-noise tunable continuous-wave laser. Here, we use the full-width at half maximum (FWHM) of observed spectral lineshapes to approximate the µOPO signal linewidth. In particular, we are interested in relative orders of magnitude between the pump laser linewidth (≈300 kHz) and µOPO linewidths, and we reserve a more comprehensive noise analysis for future studies. In Fig. 4c, we present heterodyne spectra for three µOPOs with signal frequencies near 470 THz, 504 THz, and 523 THz. Respectively, the fitted lineshapes exhibit FWHM values of ≈547 kHz, ≈482 kHz, and ≈504 kHz, where the figure includes the one standard deviation uncertainties obtained from the fits. These values are commensurate with the pump laser linewidth and demonstrate low added noise from the µOPO; moreover, they are much smaller than typical III-V diode lasers in this wavelength range. In a more absolute sense, the measured linewidths are already sufficiently small for many high-coherence applications, and future systems could employ injection locking to achieve low-noise operation even with noisy pump lasers^22,45,46^.

## Discussion

In summary, we establish a blueprint, based on widely separated Kerr OPO, for integrated sources of coherent and highly tunable light at green-gap frequencies. We generate the most widely separated µOPOs to date, with a signal frequency reaching ≈563.51 THz and its corresponding idler near ≈207.28 THz. Dispersion simulations suggest that cyan emission is possible with our scheme, but it is currently not observed due to parasitic losses in the idler band. Finally, we note that the resonator-waveguide coupling was not optimized to realize large conversion efficiencies, but we expect that conventional strategies to increase coupling (e.g., using pulley waveguide geometries^30^) will enable efficient green emission. Preliminary measurements, presented in Supplementary Information section V, indicate that engineered coupling waveguides can improve efficiency; in a pulley-coupled test device we estimate the on-chip signal power to be ≈500 µW (Supplementary Information Fig. S7), and higher power is possible after further coupling optimization^30^.

## Materials and methods

Simulations in Figs. 2 and 3 are based on eigenmode calculations of the microrings using the finite-element method. The layout of the devices is prepared using the Nanolithography Toolbox, a free software package provided by the NIST Center for Nanoscale Science and Technology^47^. A 605-nm-thick layer of SiN is deposited by low-pressure chemical vapor deposition on top of a 3 µm SiO_2_ layer on a 100 mm Si wafer. Spectroscopic ellipsometry is employed to measure the layer thicknesses and the wavelength-dependent refractive indices, and the results are fitted using an extended Sellmeier model. The device patterning is realized using positive-tone resist and electron-beam lithography, followed by pattern transfer into the SiN layer through reactive-ion etching using a CF_4_/CHF_3_ chemistry. The device undergoes chemical cleaning to remove any residual polymer or resist post-etching and is subsequently annealed at 1100 °C in a nitrogen environment for four hours. An oxide lift-off process is executed to ensure air-cladding on the devices while maintaining oxide-cladding over the input and output waveguides. The chip is then diced and polished for lensed-fiber coupling. The microring undercuts are achieved by heated KOH etching at 70 °C, with the lateral etching rate estimated by measuring the vertical etching rate and verified through cross-sectional scanning electron microscope images.

### Supplementary information

Supplementary Information

## Data Availability

The data that supports the plots within this paper and other findings of this study are available from the corresponding author upon reasonable request.
