# Supplementary material for: Advancing on-chip Kerr optical parametric oscillation towards coherent applications covering the green gap
Source: Light Sci Appl. 2024 Aug 21;13:201. doi: 10.1038/s41377-024-01534-x (PMC11339420; doi:10.1038/s41377-024-01534-x)
Supplement: Supplementary file 1 — Supplementary Information [file 41377_2024_1534_MOESM1_ESM.pdf]

Supplementary Information for Advancing on-chip Kerr optical parametric oscillation towards coherent applications covering the green gap

Yi Sun,[1,2,†] Jordan Stone,[1,2,†] Xiyuan Lu,[1,2,*] Feng Zhou,[1,2] Junyeob Song,[1] Zhimin Shi,[3] and Kartik Srinivasan[1,2,*]

[1]Microsystems and Nanotechnology Division, Physical Measurement Laboratory, National Institute of Standards and Technology, Gaithersburg, Maryland 20899, USA

[2]Joint Quantum Institute, NIST/University of Maryland, College Park, Maryland 20742, USA

[3]Reality Labs Research, Meta, Redmond, Washington 98052, USA

This document provides supplementary information for "Advancing on-chip Kerr optical parametric oscillation towards coherent applications covering the green gap", including dispersion simulations, measurement details, data and analysis concerning the most widely-separated optical parametric oscillation observed in experiments, spectra generated in the set of four devices that together cover the "green gap", continuous frequency tuning using temperature control, and preliminary power measurements from green-gap devices with engineered coupling.

I. SIMULATION ANALYSIS OF COARSE FREQUENCY TUNING FOR DIFFERENT GEOMETRIES

In the main text, we studied the relationships between resonator geometry, dispersion, and μOPO frequencies. Such information is useful to evaluate μOPO robustness with respect to fabrication uncertainties, but it does not fully convey the coarse tunability of different devices. In Fig. S1, we present simulated $\Delta\nu$ spectra, as well as simulations of ν_s versus ν_p, for five different (H, U) designs. It is clear that increasing H or U decreases the sensitivity of ν_s to ν_p changes, which allows us (given the requisite ν_p tuning) to generate more μOPOs with denser spectral coverage using a single device. Furthermore, the information in Fig. S1 can be used to design (H, U) according to case-by-case constraints, such as available ν_p tuning.

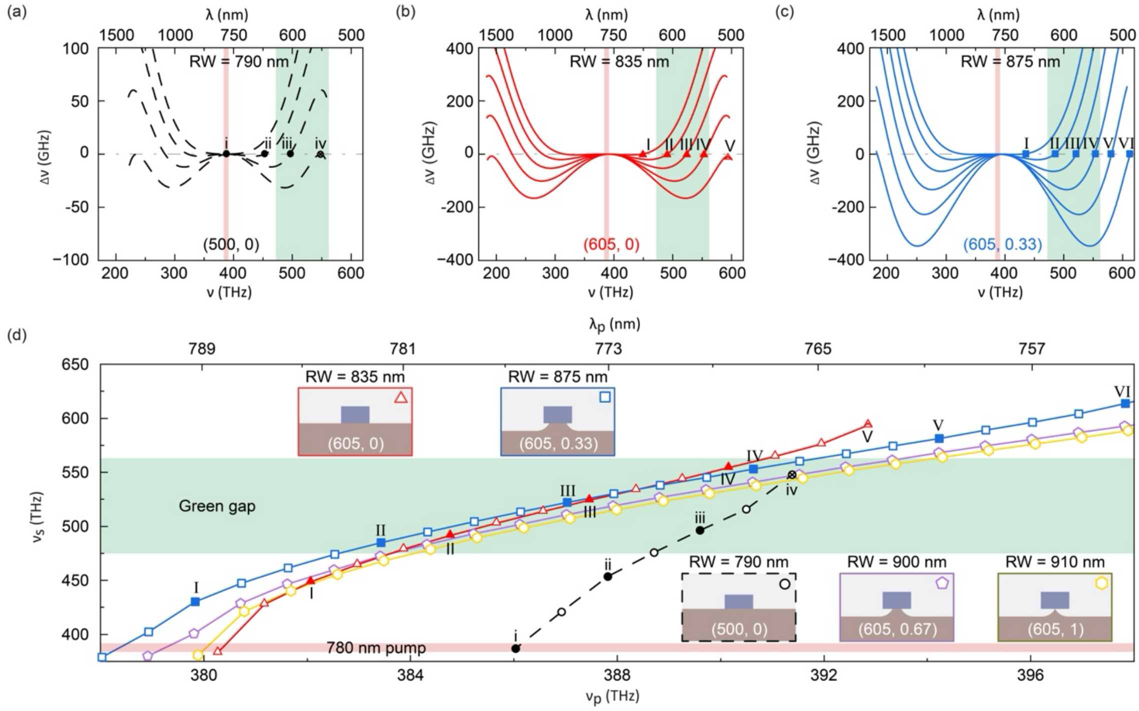

Figure S1: Study of coarse μOPO frequency tuning for different resonator geometries. **a** Simulated $\Delta\nu$ spectra for the (500, 0) design with RW = 790 nm. Different curves correspond to different ν_p values. Points marked by lower-case numerals correspond to ν_s and ν_p values in part (d). **b** Simulated $\Delta\nu$ spectra for the (605, 0) design with RW = 835 nm. **c** Simulated $\Delta\nu$ spectra for the (605, 0.33) design with RW = 875 nm. **d** Simulated values of ν_s versus ν_p for different (H, U) and RW. Points marked by crosses correspond to the upper ν_s limit.

†These two authors contributed equally.

*Electronic address: xnl9@umd.edu

*Electronic address: kartik.srinivasan@nist.gov

As another illustration of robustness, we simulate the resonator dispersion for a device with incomplete SiN etching (which corresponds more closely to experimental devices; see the FIB image in Fig. 2b of the main text) and make a comparison with a fully-etched device (i.e., with an ideal rectangular cross section). We present the simulation results in Fig. S2, including ν_s versus ν_p. These data indicate that incomplete etching does not substantially impact the dispersion, so that we can understand our experiments through our simulations of fully-etched devices.

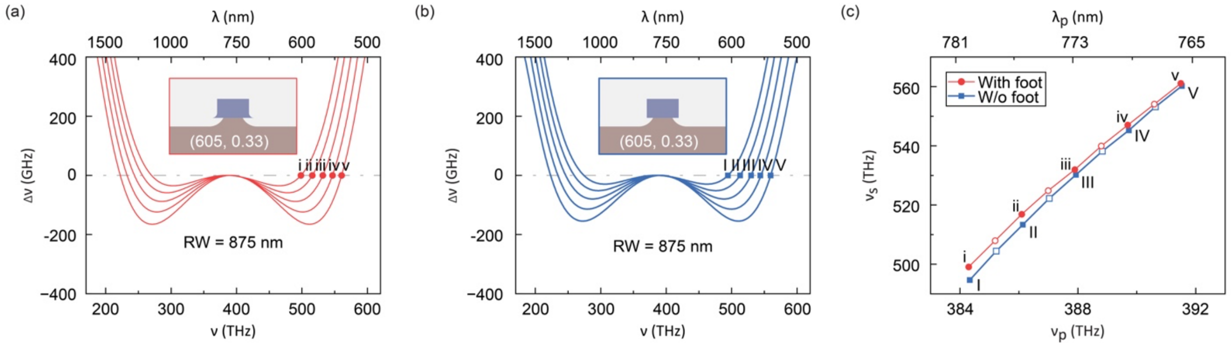

Figure S2: **a** Simulated Δν spectra for the (605, 0.33) design with RW = 875 nm. The RW is larger at the bottom to model the incomplete etching observed in our experimental devices. Different curves correspond to different ν_p values, indicated in part (c). **b** Simulated Δν spectra for the completely-etched, (605, 0.33) design with RW = 875 nm. **c** Simulated values of ν_s versus ν_p for the two geometries in (a) and (b).

II. MEASUREMENT SETUP FOR WIDELY-SEPARATED OPTICAL PARAMETRIC OSCILLATION (OPO)

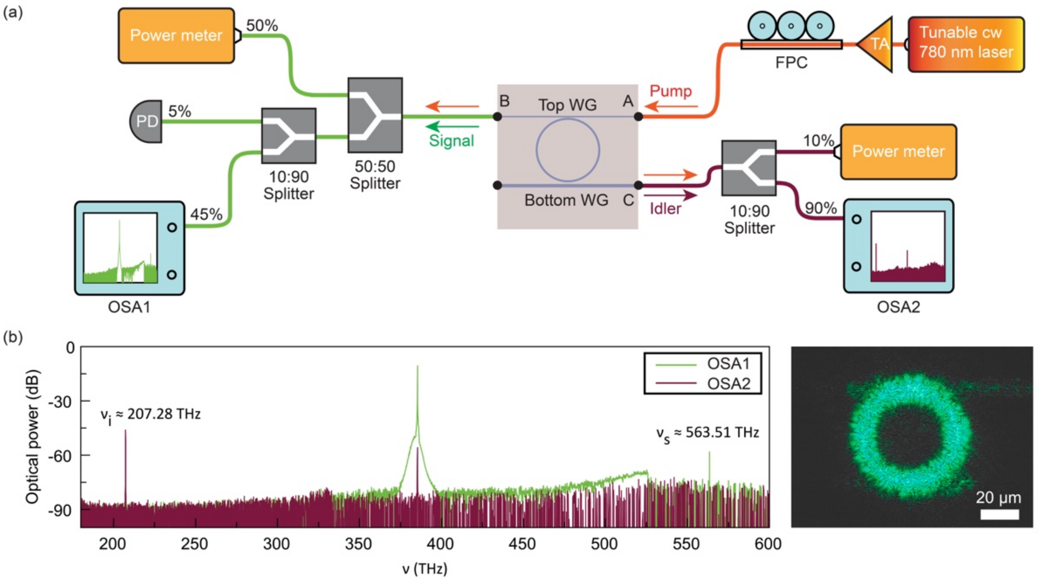

Figure S3: **a** Schematic of our measurement setup. PD: Photodiode, OSA: Optical spectrum analyzer, TA: Tapered amplifier, FPC: Fiber polarization controller. **b** Left panel: OPO spectrum exhibiting the largest signal-idler separation observed in our study. The green spectrum indicates the signal and pump waves and is captured from the top WG using OSA1. The idler is not visible because it cannot propagate in the top WG. The dark red spectrum indicates the idler and pump waves and is captured from the bottom WG using OSA2. The signal is significantly undercoupled to the bottom WG; hence, it is not detectable. Right panel: Optical microscope image of the microring during OPO. Scattered signal light (green) is clearly visible.

Figure S3a illustrates our measurement setup for OPO experiments. We use lensed fibers to in/out-couple light to/from inverse tapered waveguides (WGs). The estimated insertion loss for the pump is ≈3 dB per facet. The top WG is employed for in- and out-coupling the pump and signal waves, while the idler is extracted from the bottom WG. We use this configuration for both linear cavity transmission measurements (as shown in Fig. 3a of the main text) and high-power OPO measurements. For linear cavity transmission measurements, we use a continuous-wave (CW) laser whose wavelength is tunable from 765 nm 781 nm. We attenuate the laser power to sub-microwatt levels and adjust its polarization to overlap the microring TE modes. In the case of OPO measurements, we can boost the pump laser power

up to ≈300 mW using a tapered amplifier, while most of the measurements are performed with ≈ 100 mW. The on-chip pump power is estimated ≈ 50 mW considering ≈ 3 dB facet coupling loss for the pump. Spectra are obtained with two optical spectrum analyzers (OSAs). OSA1 collects light from the top WG and OSA2 collects light from the bottom WG, as shown in Fig. S1a. To illustrate the need for two WGs, in Fig. S1b we present the OPO spectrum with the widest signal-idler separation observed in our study. The signal frequency is $\nu_s \approx 563.51$ THz, which corresponds to an idler frequency $\nu_i \approx 207.28$ THz. In the spectrum extracted from the top WG, we detect (do not detect) measurable amounts of signal (idler) power, and vice versa. The right panel displays a microscope image of the microring in which scattered signal light is clearly visible.

III. KERR OPO COVERING THE GREEN GAP

In Fig. 4a of the main text, we show a set of overlaid optical spectra compiled from four devices. Here, we separate the spectra and sort them according to which device was used to generate them; see Fig. S4. We note, in the two devices indicated by square and circle symbols, OPO spectra via pumping of eight different pump modes, covering the spectral range from ≈ 490 THz to ≈560 THz with > 100 total spectra. For devices marked with a star or diamond, we needed only to pump one or two modes to target the remaining portion of the green gap, from ≈ 475 THz to ≈490 THz. Therefore, the total span of signal wavelengths accessible using these four devices is much greater than we present, though here we obviously focus on the green gap.

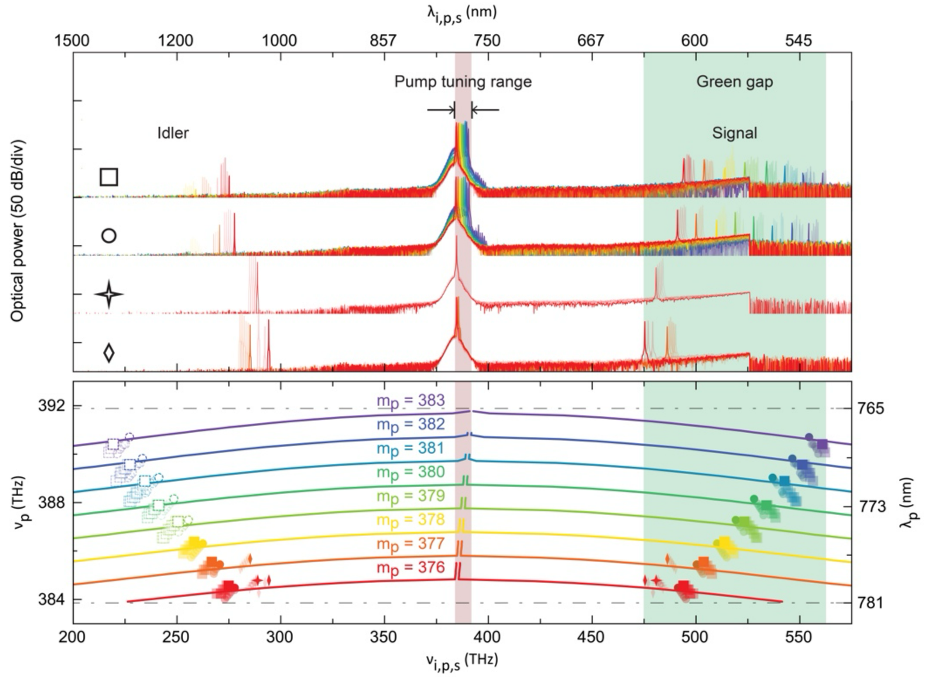

Figure S4: Top panel: Compilation of optical spectra generated from four devices. The different colors correspond to the mp values as marked in the bottom panel. The bold data correspond to the first OPO spectrum observed when the pump laser is blue-to-red scanned through a given pump mode. The faded data correspond to subsequently observed OPO spectra (the same as in Fig. 3c in the main text). Idlers with frequencies below 250 THz are not observed due to the pump/signal waveguide cutoff. Bottom panel: Distribution of the signal and idler frequencies from these four devices as extracted from the spectra above. Solid lines are taken from dispersion simulations.

IV. CONTINUOUS TUNING WITH TEMPERATURE VARIATION

In the main text, we demonstrate continuous ν_s tuning up to 80 GHz using ν_p actuation. Here, we show one route to extend the tuning range using temperature variation. We change the temperature of our sample by heating (using a resistive strip heater) the metal mount on which it sits. In Fig. S5a, we present measurements of ν_s (recorded using a wavemeter) versus ν_p for different temperature setpoints ranging from 27 degrees to 85 degrees Celsius. As ν_p is tuned, we observe continuous ν_s tuning with intermittent mode hops, as described in the main text. However, for different temperatures, the structure of these mode hops is altered, increasing the range of accessible ν_s values to upwards of 200 GHz in some cases. In Figs. S5b-c, we have zoomed into a narrower range of ν_s values to establish that our tuning is truly continuous and reversible.

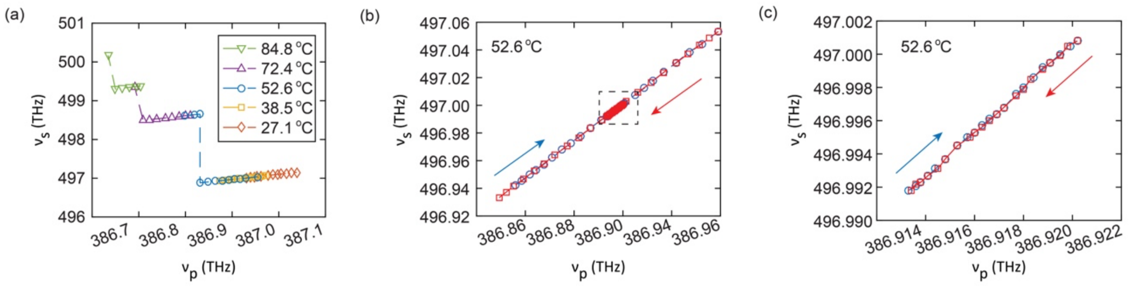

Figure S5: **a** Wavemeter measurements of the OPO signal frequency, ν_s, versus pump frequency, ν_p, for different temperature setpoints in a nominal device. **b** Higher-resolution measurement of ν_s versus ν_p at a temperature setpoint of 52.6 degrees Celsius. **c** Same measurement zoomed into the region indicated by the dashed box in (b).

V. IMPROVING SIGNAL EXTRACTION EFFICIENCY

For devices studied in the main text, we use a top WG (see Fig. S3a) coupling gap of 100 nm, which corresponds to near-critical waveguide-resonator coupling at the pump wavelength, but necessarily leaves the OPO signal undercoupled. Such undercoupling is responsible for the relatively small amounts of extracted signal power measured in the main text. To prove that greater green-gap extraction efficiencies are possible, we here demonstrate two promising experiments. In the first, as shown in Fig. S6a, we introduce a bottom WG with a coupling gap of 70 nm that is designed to be cut-off for pump wavelengths and near critically coupled for signal wavelengths (instead of the idler, as in our previous experiments). In this configuration, OSA1 collects light from the top WG and OSA2 collects light from the bottom WG. We pump a μOPO with $\nu_s \approx 555$ THz. Green light is more efficiently extracted to the bottom WG, as confirmed by the microscope image, and OSA measurements indicate that the bottom WG is more than ten times better at extracting green light than the top WG, as shown in Fig. S6b. Furthermore, we calibrate the optical losses during propagation from the bottom WG to OSA2 to be ≈25 dB. Hence, we estimate the on-chip signal power for this OPO device to be ≈100 μW. Such powers are already sufficient for many applications and demonstrate, in principle, that optimized coupling configurations greatly improve efficiency.

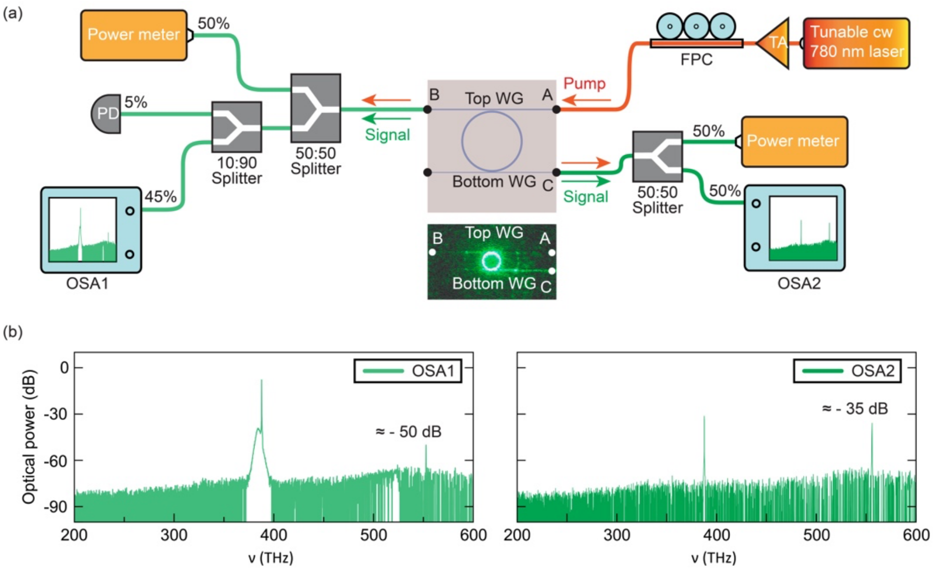

Figure S6: **a** Measurement setup for μOPO devices using a separate bottom WG for signal extraction. **b** OPO spectrum recorded using both OSA1 (left panel) and OSA2 (right panel). The optical power extraction is ≈15 dB more efficient in the bottom WG than the top WG. Here, 0 dB is referenced to 1 mW, i.e., dBm. After calibration of losses, the on-chip signal power in the bottom WG is estimated to be ≈100 μW.

In the second experiment, as shown in Fig. S7a, we implement a single 3 μm pulley waveguide[1] to couple both pump light and signal light at redder wavelengths within the green gap. We pump a μOPO with $\nu_s \approx 515$ THz and present the optical spectrum in Fig. S7b. We note that signal light appears more powerful than pump light in this spectrum due to wavelength-dependent losses in the path between the chip and OSA. Nonetheless, after calibrating for such losses, we

calculate the on-chip signal power to be ≈ 500 μW for this μOPO, and this power level is maintained during ν_s tuning, as shown in Fig. S7c. Moreover, the output powers and efficiencies we observe compare favorably to demonstrations of integrated THG[2,3]. In the future, we expect further coupling optimization (e.g., through use of drop-port pulley geometries) to enable milliwatt-level on-chip signal powers.

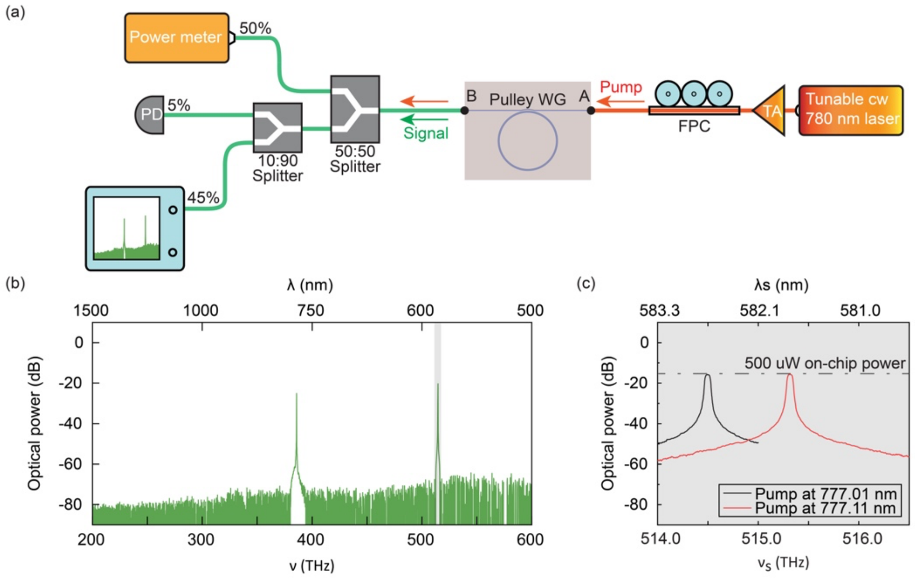

Figure S7: **a** Measurement setup for μOPO devices using one single pulley top WG for signal extraction. **b** OPO spectrum recorded using the OSA. **c** Zoomed-in OPO spectrum showing that signal power is maintained while tuning ν_p. Here, 0 dB is referenced to 1 mW, i.e., dBm. After calibration of losses, the on-chip signal power is estimated to be ≈ 500 μW.

Supplementary information accompanies the manuscript on the Light: Science & Applications website (http://www.nature.com/lsa)

References

1 Stone, J. R. et al. Efficient chip-based optical parametric oscillators from 590 to 1150 nm. *APL Photonics* **7**, 121301 (2022).

2 Levy, J. S. et al. Harmonic generation in silicon nitride ring resonators. *Optics Express* **19**, 11415–11421 (2011).

3 Surya, J. B. et al. Efficient third-harmonic generation in composite aluminum nitride/silicon nitride microrings. *Optica* **5**, 103–108 (2018).
